# A retrospective study of alectinib versus ceritinib in patients with advanced non–small-cell lung cancer of anaplastic lymphoma kinase fusion in whom crizotinib treatment failed

**DOI:** 10.1186/s12885-021-08005-1

**Published:** 2021-03-24

**Authors:** Chih-Hsi Scott Kuo, Pi-Hung Tung, Allen Chung-Cheng Huang, Chin-Chou Wang, John Wen-Cheng Chang, Chien-Ying Liu, Fu-Tsai Chung, Yueh-Fu Fang, Yi-Ke Guo, Cheng-Ta Yang

**Affiliations:** 1 Division of Thoracic Oncology, Department of Thoracic Medicine, Chang Gung Memorial Hospital, Chang Gung University, College of Medicine, Taoyuan City, Taiwan; 2 Department of Medical Oncology, Chang Gung Memorial Hospital, Chang Gung University, Taoyuan City, Taiwan; 3 Department of Computing, Imperial College London, Data Science Institute, London, UK; 4 Division of Pulmonary & Critical Care Medicine, Kaohsiung Chang Gung Memorial Hospital, Kaohsiung, Taiwan

**Keywords:** NSCLC, ALK, Crizotinib, Treatment failure, Alectinib, Ceritinib, CNS

## Abstract

**Background:**

Crizotinib is the approved treatment for advanced non-small cell lung cancers (NSCLCs) of anaplastic lymphoma kinase (ALK) fusion. Failure of crizotinib treatment frequently involves drug intolerance or resistance. Comparison of using second-generation ALK inhibitors in this setting remains lacking.

**Methods:**

Sixty-five ALK-positive advanced NSCLC patients receiving second-generation ALK inhibitors following treatment failure of crizotinib were retrospectively analyzed for the therapeutic efficacy.

**Results:**

Forty-three (66.2%) and 22 (33.8%) patients received alectinib and ceritinib, respectively. Comparing alectinib to ceritinib treatment: the 12-month progression-free survival (PFS) rate (61.0% [95% confidence interval, 47.1 to 78.9%] vs. 54.5% [95% CI, 37.3 to 79.9%]); the hazard ratio (HR) for disease progression or death, 0.61 (95% CI, 0.31–1.17; *p* = 0.135). Multivariate Cox regression showed ECOG PS (0–1 vs. 2–3 HR 0.09 [95% CI, 0.02–0.33]; *p* < 0.001) and cause of crizotinib treatment failure (resistance vs. intolerance HR 2.75 [95% CI, 1.26–5.99]; *p* = 0.011) were the independent predictors for the PFS of second-generation ALK inhibitors. Treatment of alectinib, compared to ceritinib, was associated with a lower incidence of CNS progression (cause-specific HR, 0.10; 95% CI 0.01–0.78; *p* = 0.029) and a higher efficacy in patients whose cause of crizotinib treatment failure was intolerance (HR 0.29 [95% CI, 0.08–1.06]; *p* = 0.050). The most commonly noted adverse events were elevated AST/ALT in 10 (23.3%) patients treated with alectinib and diarrhea in 8 (36.4%) patients treated with ceritinib.

**Conclusion:**

Second-generation ALK inhibitors in crizotinib-treated patients showed a satifactory efficacy. Alectinib treatment demonstrated a CNS protection activity and a higher PFS in selected patients failing crizotinib treatment.

## Key point

1. Second-generation ALK inhibitors produced a favourable efficacy in a cohort of crizotinib-treated ALK-positive advanced NSCLC patients.
2. The efficacy of second-generation ALK inhibitors was higher in patient whose crizotinib treatment failure was due to intolerance than due to resistance.
3. Compared with ceritinib treatment, alectinib treatment demonstrated a higher CNS protection activity and a higher PFS for selected crizotinib-treated patients.

## Background

ALK-fusion oncogenic driver accounts for the tumor development in 3–5% of patients with lung adenocarcinoma [1, 2]. The therapeutic strategy that targets this oncogenic fusion has greatly improved the prognosis of patients with advanced and metastatic disease, evidenced by an unprecedented 5-year survival rate of approximately 50% in ALK-positive NSCLC patients treated with an ALK inhibitor [3, 4].

The first-generation ALK inhibitor crizotinib was approved by the U.S. Food and Drug Administration as a standard of care for advanced ALK-positive NSCLCs in 2011, and in 2014, it was proven to be superior to the platinum-based chemotherapy as the front-line treatment [5]. However, a numbers of inherent pharmacologic properties may give rise to the treatment failure of crizotinib. In terms of the kinase selectivity at a clinical relevant dose level, crizotinib suppresses not only ALK but also MET and ROS1 and it demonstartes a low probability of suppressing RON and AXL kinase [6]. Consequently, crizotinib treatment leads to more adverse effect-related dose reduction and discontinuation than treatment with newer generation ALK inhibitors [7, 8].

Crizotinib possessed a lower capacity of ALK inhibition in vitro than other newer generation ALK inhibitors [9, 10] and its concentration at tumour sites may be influenced by the drug transporter P-glycoprotein [11] which is present in a wide range of human tissues including the blood-brain barrier [12], liver and adrenal gland [13]. Consequently, disease progression related to inadequate kinase suppression may also account for crizotinib treatment failure. Hot spot mutation of the ALK kinase domain, a pivotal drug resistance mechanism, plays a less significant role in contributing to the failure of crizotinib treatment in the front-line setting [14]. Thus, crizotinib is currently a less recommended agent for the front-line treatment compares to the other newer generation ALK inhibitors.

Regardless to the cause of crizotinib treatment failure, subsequent treatment with second-generation ALK inhibitors is preferred over chemotherapy [15]; as earlier study had indicated that sequential ALK inhibitor administration was a more favourable course that yielded a longer overall survival than a course of crizotinib followed by chemotherapy [3]. Previous study on ceritinib treatment of crizotinib-pretreated patients demonstarted a 45–55% response rate and a 5–7 month PFS [16, 17]. Treatment with alectinib in a similar setting also yielded a 40–50% response rate and a 7–8 month PFS [18, 19]. When brigatinib was administered to crizotinib-pretreated patients at 90 and 180 mg, a PFS of 9.2 and 16.7 months were obtained respectively [20]. Next generation sequencing for the study of ALK mutations may not be imperative to guide the prescription of a second-generation ALK inhibitor in such circumstances [17]; as earlier studies had revealed that the response to the second- generation ALK inhibitors was independent of the presence of an ALK kinase domain mutation [21].

At the meantime, no randomized comparison of the treatment efficacy between different second-generation ALK inhibitors in crizotinib-pretreated patients has been conducted, except an ongoing ALTA-3 trial that compared alectinib and brigatinib among patients with disease progression after crizotinib treatment [22]. Additionally, in real-world practice, the analysis of therapeutic efficacy of second-generation ALK inhibitors by the cause of failure of previous crizotinib treatment has not been reported. Therefore, in present study, we analysed the treatment efficacy of ceritinib and alectinib in a group of ALK-positive patients who underwent treatment failure with crizotinib. The efficacy of ceritinib and alectinib in terms of resistance or intolerance to the previous crizotinib treatment was also analysed.

## Methods

### Patients and treatment

We retrospectively reviewed and included 65 patients of advanced or metastatic NSCLC patients who: (1) were diagnosed of ALK fusion by Ventana ALK (D5F3) CDx immunohistochemistry assay (Roche Diagnostics, USA) in Chang Gung Memorial Hospital between January 2016 and May 2018. (2) Received subsequent treatment of alectinib 600 mg twice daily or ceritinib 750 mg daily after treatment failure of crizotnib. Patients who had tumour recurrence after curative surgery or received radiotherapy for non-palliative purpose were excluded. The progression-free survival (PFS) was defined as the interval between the date of starting alectinib or ceritinib and the date of radiologically documented progression or death. The treatment response, including complete response (CR), partial response (PR), stable disease, and progressive disease, was evaluated according to the Response Evaluation Criteria in Solid Tumors (version 1.1). The pattern of post-alectinib or post-ceritinib disease progression were also reviewed and defined as either systemic progression without prior CNS progression/death or CNS progression without prior systemic progression/death as earlier described [23]. The recording of toxicity profiles for alectinib or ceritinib treatment was performed by systemic chart review and toxicity was graded according to the National Cancer Institute Common Toxicity Criteria, version 5.0. The study used data from the Chang Gung Research Database and the study protocol was approved by the Ethics Committee of Chang Gung Memorial Hospital.

### Statistical analysis

The Mann-Whitney test was used to determine the statistical significance of continuous variables between the two groups and Fisher exact test was used for evaluating the categorical variables. The Kaplan-Meier survival curve was analysed using the R package *survival*, and the hazard ratio (HR) was analysed using the Cox regression model. The post-alectinib or post-ceritinib disease progression patterns were treated as competing risk events of which the cumulative incidence functions were calculated [24]. The modified Cox regression model for the subdistribution hazard of the cumulative incidence function was applied to calculate the disease progression hazard from a given pattern in the presence of competing events by using the R package *cmprsk* [25]. The propensity-score-matched analysis was used to balance the clinical characteristics between the treatment groups. Briefly, the alectinib and ceritinib groups served as the dependent variables and the covariates used included age, brain metastasis and prior chemotherapy. The pairs of alectinib and ceritinib individuals with equivalent propensity scores were selected in a 1:1 manner using the R package *MatchIt*. All the reported *p* values were two sided, and a *p* < 0.05 was considered statistically significant. Data were also analysed using SPSS (version 10.1; SPSS, Chicago, IL, USA).

## Results

### Baseline patient characteristics

Of the 65 patients with ALK-positve NSCLC who underwent treatment failure of crizotinib, 43 (66.2%) received alectinib and 22 (33.8%) received ceritinib as the subsequent treatment. The baseline characteristics between the alectinib and the ceritinib groups are shown in Table 1. Most features were well-balanced between the two groups, including age, sex, performance status, histology and presence of brain metastasis. The crizotinib treatment failure cause, resistance or intolerance, did not differ between the two groups. More patients received prior chemotherapy in the ceritinib group (14; 63.6%) than in the alectinib group (12; 27.9%; *p* = 0.012, Table 1). The median follow-up duration was 16.8 months and 32.0 months in the alectinib and ceritinib groups, respectively. The longer median follow-up time in ceritinib group is because ceritinib was approved 14 months earlier than alectinib for the treatment of ALK-positive NSCLC in Taiwan.

**Table 1:** Baseline characteristics of the study population

Variables, n (%)	Alectinib (***n*** = 43)	Ceritinib (***n*** = 22)	***p***-value
**Age, median (range), year**	62 (48 ~ 66)	57 (54 ~ 74)	0.501
**Sex**			
male	19 (44.2)	9 (40.9)	1.000
female	24 (55.8)	13 (59.1)	
**Smoking history**			
Smoker/ex-smoker	8 (18.6)	2 (9.1)	0.520
Nonsmoker	35 (81.4)	20 (90.9)	
**ECOG PS**			
0 /1	40 (93.0)	20 (90.9)	1.000
2/ 3	3 (7.0)	2 (9.1)	
**Histology**			
Adenocarcinoma	43 (100.0)	22 (100.0)	1.000
**Brain metastasis**			
Yes	13 (37.2)	11 (50.0)	0.426
No	30 (62.8)	11 (50.0)	
**Cause of crizotinib treatment failure**			
Resistance	26 (60.5)	14 (63.6)	1.000
Intolerance	17 (39.5)	8 (36.4)	
**Prior chemotherapy**			
Yes	12 (27.9)	14 (63.6)	0.012
No	31 (72.1)	8 (36.4)	

*ECOG PS* Eastern Cooperative Oncology Group performance status

### Treatment efficacy between alectinib and ceritinib

**Fig. 1:**
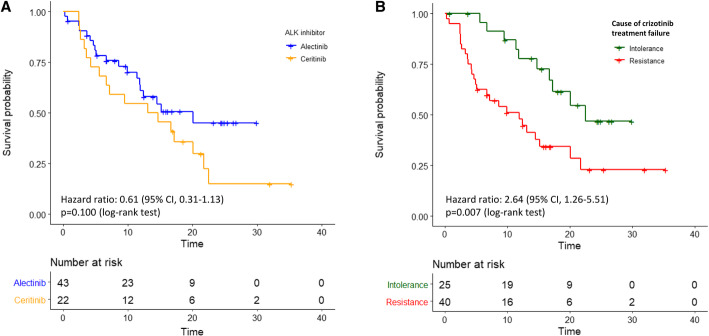
(**a**) PFS between alectinib and ceritinb treatment groups (**b**) PFS between the treatment groups in terms of crizotinib treatment failure patterns

At the time of analysis, 19 (44.2%) events of disease progression or death were noted in the alectinib group and 17 (77.3%) events were noted in the ceritinib group. Patients receiving alectinib treatment, compared to ceritinib, showed a similar 12-month PFS rate (61.0% [95% confidence interval, 47.1 to 78.9%] vs. 54.5% [95% CI, 37.3 to 79.9%]); HR for disease progression or death, 0.61 (95% CI, 0.31–1.17; *p* = 0.135) and median PFS (20.1 vs. 13.9 months; log-rank test *p* = 0.100, Fig. 1a) than those receiving ceritinib treatment. The tumor response was estimable in 63 patients (41 treated with alectinib and 22 treated with ceritinib), with the CR and PR being 2.4 and 70.8%,respectively, in the alectinib group and the PR being 50% in the ceritinib group. A numerically higher response rate was noted in the patients who received alectinib treatment (73.2 vs. 50.0%, *p* = 0.096; Table 2).

**Table 2:** Objective response in the study population

Variables, n (%)	Alectinib (***n*** = 41)^a^	Ceritinib (***n*** = 22)
**Response**		
No. of patients	30	11
% (95% CI)	73.2 (57.1–85.8)	50.0 (28.2–71.8)^#^
**Complete response--** no. (%)	1 (2.4)	0
**Partial response--**no. (%)	29 (70.8)	11 (50.0)
**Stable disease**--no. (%)	6 (14.6)	6 (27.3)
**Progression disease--**no. (%)	5 (12.2)	5 (22.7)

^a^Not evaluable in 2 patients. # p = 0.096 for the comparison between alectinib and ceritinib

### Analysis of predictors for treatment efficacy in all patients

**Fig. 2:**
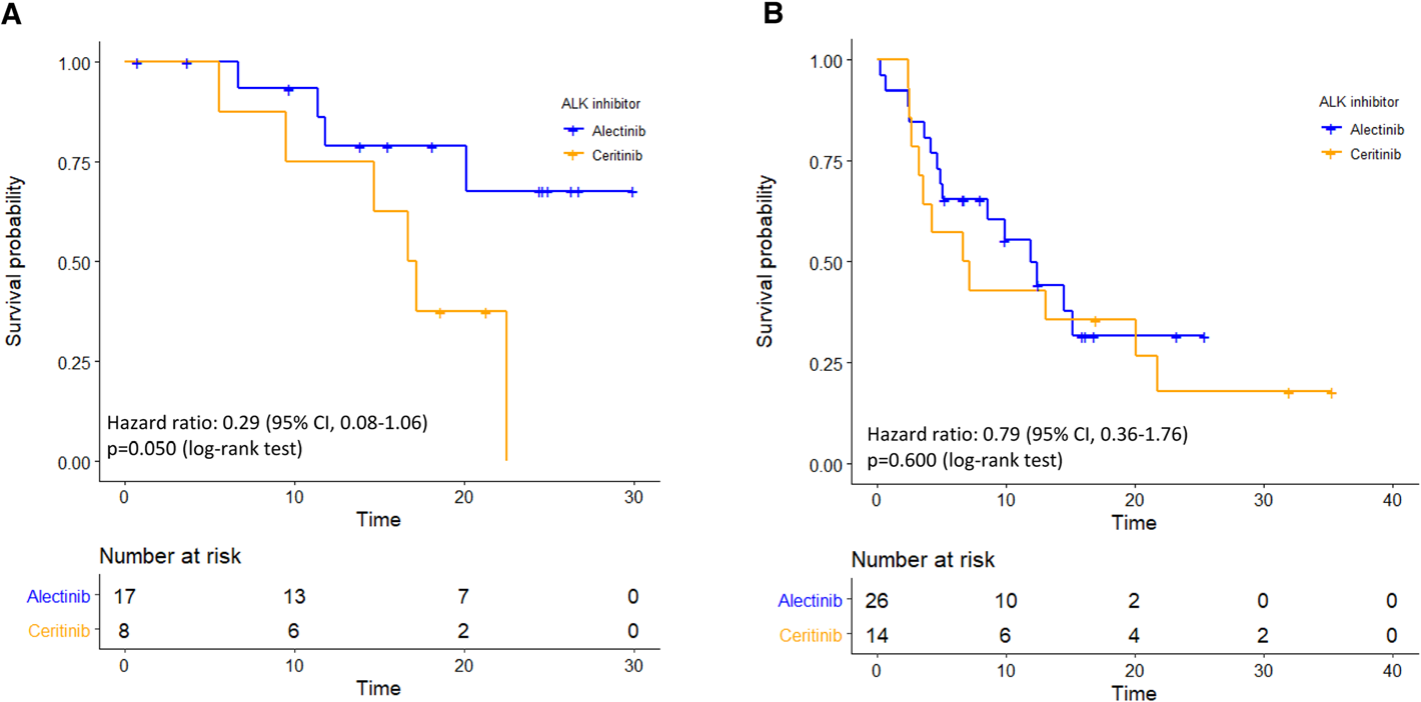
PFS between alectinib and ceritinb in (**a**) subgroup of patients of crizotinib treatment failure due to intolerance (17 patients received alectinib and 8 patients received ceritinib in which 4 and 6 events were observed, respectively) and in (**b**) subgroup of patients of crizotinib treatment failure due to resistance (26 patients received alectinib and 14 patients received ceritinib in which 16 and 11 events were observed, respectively)

**Fig. 3:**
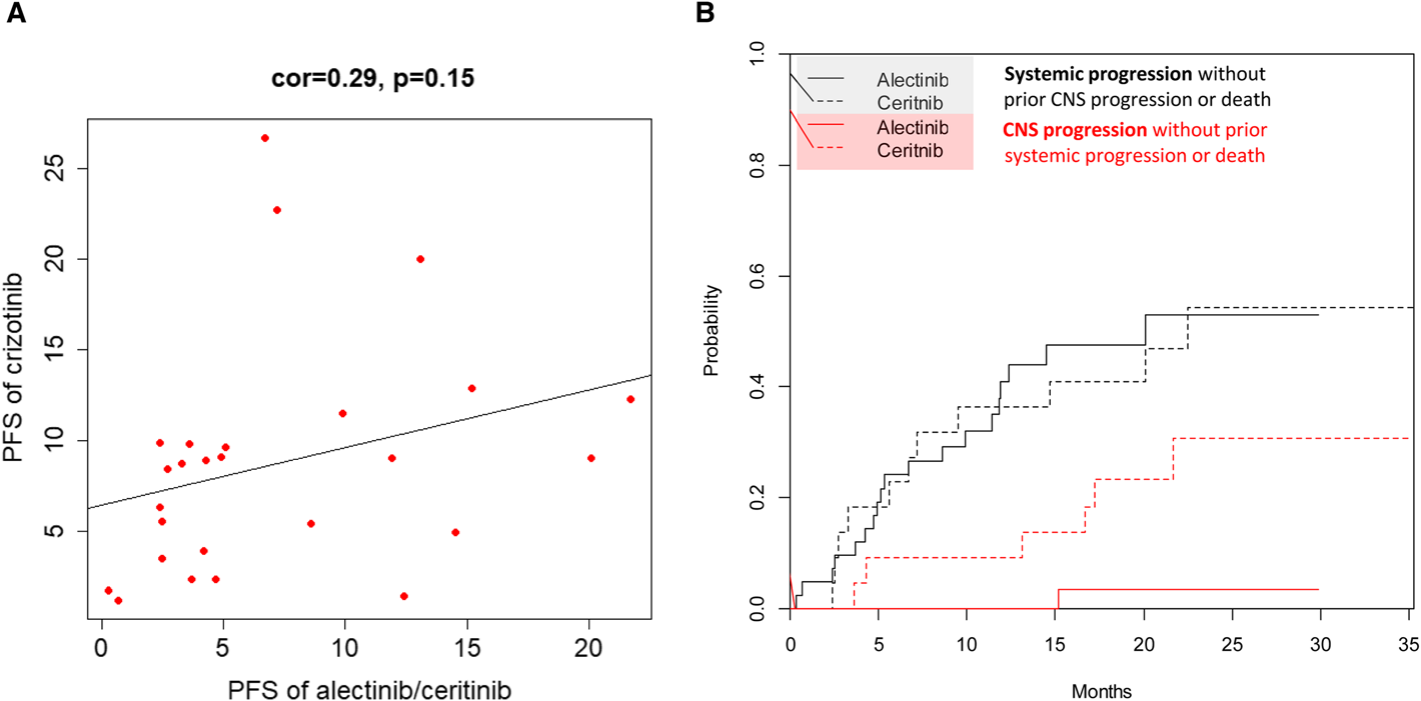
**a** The relationship between PFS of crizotinib and subsequent alectinib/ceritinib in patients who underwent drug resistance in the two lines of treatment. **b** Cumulative incidence of systemic progression (black) and CNS progression (red) between the alectinib (solid line) and ceritinib (broken line) treatment

The clinical predictors associated with the PFS were analysed in all patients. In univariate Cox regression; ECOG PS 0–1 (HR 0.10 [95% CI, 0.03–0.29]; *p* < 0.001) and alectinib treatment (HR 0.61 [95% CI, 0.31–1.13]; *p* = 0.135; Table 3) were associated with a longer PFS. By contrast, brain metastasis (HR 1.61 [95% CI, 0.82–3.17]; *p* = 0.119) and crizotinib treatment failure due to resistance (HR 2.64 [95% CI, 1.26–5.51]; *p* = 0.009; Table 3**and** Fig. 1b) were associated with a reduced PFS. Prior chemotherapy (HR, 1.18 [95% CI, 0.61–2.27]; *p* = 0.630) had no impact on the PFS. In the multivariate analysis, ECOG PS 0–1 (HR 0.09 [95% CI, 0.02–0.33]; *p* < 0.001) and crizotinib treatment failure due to resistance (HR 2.75 [95% CI, 1.26–5.99]; *p* = 0.011; Table 3) remained the independent and significant predictors of the PFS. We further examined the treatment efficacies of alectinib and ceritinib by the cause of crizotinib treatment failure. In patients who discontinued crizotinib due to intolerance, the subsequent alectinib treatment improved the PFS compared with ceritinib (HR 0.29 [95% CI, 0.08–1.06]; *p* = 0.050, Fig. 2a). However, in patients who discontinued crizotinib due to resistance, the efficacy was similar between the subsequent alectinib and ceritinib treatment (HR 0.79 [95% CI, 0.36–1.76]; *p* = 0.600, Fig. 2b). In this group of patients who underwent disease progression on alectnib/ceritinib treatment, a weakly positive correlation between the PFS of crizotinib and alectnib/ceritinib was observed (Pearson’s correlation r = 0.29, *p* = 0.150; Fig. 3a).

**Table 3:** Cox regression analysis of the progression-free survival

	Univariate		Multivariate	
Variables	HR (95% C.I.)	p-value	HR (95% C.I.)	p-value
**Age**	0.99 (0.96–1.01)	0.344	–	–
**Sex (male)**	1.04 (0.53–2.04)	0.751	–	–
**Smoking history**	0.79 (0.28–2.23)	0.608	–	
**ECOG PS 0–1**	0.10 (0.03–0.29)	< 0.001	0.09 (0.02–0.33)	< 0.001
**Brain metastasis**	1.61 (0.82–3.17)	0.119	1.22 (0.58–2.56)	0.594
**Cause of crizotinib treatment failure: Resistance**^a^	2.64 (1.26–5.51)	0.009	2.75 (1.26–5.99)	0.011
**Prior chemotherapy**	1.18 (0.61–2.27)	0.630	–	–
**Alectinib vs. ceritinib**	0.61 (0.31–1.13)	0.135	0.68 (0.33–1.37)	0.277

*ECOG PS* Eastern Cooperative Oncology Group performance status; ^a^ as opposed to crizotinib intolerance

### Disease progression pattern between alectinib and ceritinib

The disease progression pattern after alectinib and ceritinb treatment was analysed, in terms of the cumulative incidence of systemic or CNS progression. The rate of CNS progression with time was significantly lower after alectinib treatment than after ceritinib treatment (cause-specificHR, 0.10; 95% CI 0.01–0.78; *p* = 0.029, Fig. 3b), and 1 (2.3%) patients in the alectinib group and 6 (27.3%) patients in the ceritinib group reported an event of CNS progression. The rate of systemic progression did not differ between the alectinib and ceritinib groups over time (cause-specific HR, 1.04; 95% CI 0.50–2.16; *p* = 0.920, Fig. 3b).

### Adverse events profile

Among patients who received alectinib treatment, the most commonly noted all-grade adverse events were elevated AST/ALT levels in 10 (23.3%) patients, myalgia in 5 (11.6%) patients and nausea in 4 (9.3%) patients. Among patients who received ceritinib treatment, the most commonly noted all grade adverse events were diarrhoea in 8 (36.4%) patients, nausea in 6 (27.3%) patients and vomiting in 4 (18.2%) patients. The most frequently noted serious adverse event associated with alectinib treatment was elevation of AST/ALT in 3 (6.9%) patients, whereas the most frequently noted serious adverse event associated with ceritinib treatment were diarrhoea in 3 (13.6%) patients and nausea in 2 (9.1%) patients. Dose reduction was required in 3 (6.9%) and 5 (22.7%) patients who received the alectinib and ceritinib treatment, respectively. Adverse event-related treatment discontinuation was noted in 1 (2.3%) patient who received alectinib treatment and none who received ceritinib treatment (Table 4).

**Table 4:** Treatment-related adverse events

Frequency n (%)	Alectinib (n = 43)		Ceritinib (n = 22)	
	Any Grade	Grade 3–5	Any Grade	Grade 3–5
Nausea	4 (9.3)	1 (2.3)	6 (27.3)	2 (9.1)
Diarrhea	2 (4.7)	0	8 (36.4)	3 (13.6)
Vomiting	3 (6.9)	0	4 (18.2)	1 (4.5)
Elevation of AST/ALT	10 (23.3)	3 (6.9)	3 (13.6)	1 (4.5)
Peripheral edema	2 (4.7)	0	2 (9.1)	0
Blurred vision	1 (2.3)	0	0	0
Myalgia	5 (11.6)	1 (2.3)	0	0
Dose reduction	3 (6.9)		5 (22.7)	
Treatment discontinuation	1 (2.3)		0	

*AST* aspartate transaminase; *ALT* alanine transaminase

## Discussion

This study analyzed the treatment efficacies of ceritinib and alectinib in ALK-positive NSCLC patients pretreated with crizotinib. The treatment efficacy of alectinib and ceritinib was similar among patients in whom crizotinib treatment failed due to resistance. However, alectinib treatment showed an improved efficacy among patients in whom crizotinib treatment failed due to intolerance and it was associated with a lower incidence of CNS progression. The major adverse events were elevated liver function in the alectinib group and gastrointestinal toxicity in the ceritinib group, respectively.

Because of a broad kinase suppression profile, administration crizotinib frequently involved adverse event-related dose modification during the treatment courses. In the global ALEX study, 21 and 25% of crizotinib-treated patients had undergone a dose reduction and interruption, respectively [8]. The dose modification frequency was even higher in the Japanese ALEX study, in which 67% of the crizotinib-treated patients required a dose reduction and 23% of them eventually withdrew from the treatment [7]. In this analysis, we observed that 38% of our crizotinib-treated patients, in a real-world setting, discontinued the treatment due to intolerance. The median duration of crizotinib treatment in these patients was 1.9 (1.2–5.7) months during which the dose modification measures had usually been taken. However, physician-judged treatment switches to a second-generation ALK inhibitor without dose modification were also observed mainly due to the wariness about tissue concentration and crizotinib activity at a reduced dose level.

Thereafter, when ceritinib or alectinib were given subsequently, these second-generation ALK inhibitors obviously produced a longer PFS than they were given with crizotinib resistance. Notably, an improved treatment efficacy of alectinib was found in these patients stopping crizotinib due to intolerance. This finding may be associated with the higher gastrointestinal toxicity presented by ceritinib and thereby more frequent dose interruption as observed previously in the ASCEND-4 study [26]. Moreover, because ceritinib is less brain penetrant than is alectinib [11], the resulting dose interruptions and insufficient serum concentration may have compromised the control of brain metastasis. This assumption was echoed in the present study where the incidence of CNS progression over time was significantly higher in patients treated with ceritinib than in those treated with alectinib. Nevertheless, as the potency of ceritinib remained assured [10], the incidence of systemic progression between the two ALK inhibitors was similar in this analysis. Recently, the gastrointestinal toxicity of ceritinib has been shown to be greatly reduced at lower doses when administered with food, without compromising on the treatment efficacy [27, 28]. Whether this dosing scheme also yielded an optimal CNS control remained unclear.

On the other hand, this analysis demonstrated that when ALK-positive patients received second-generation ALK inhibitors due to crizotinib resistance; the difference in the treatment efficacy between the two drugs was nonsignificant.. Compare to the earlier global phase III studies of the second-line ceritinb and alectinib treatment in which a 6–9 month PFS were reported [16–19]; we observed a similar 7–11 month PFS in this study of Asian ethnicity. This finding suggested that while the sequential use of second-generation ALK inhibitors may successfully addressed deficiencies about the potency and tissue concentration of crizotinib; multiple factors leading to drug resistance can shortly come into play including ALK kinase domain solvent-front, gatekeeper and compound mutations [14, 29] and the emergence of ALK independent tumor clones that conferred non-ALK resistance mechanisms [30, 31].

The present analysis had inherent limitations of the retrospective nature of study and the small sample size, while it remained valuable as the randomized comparison of the efficacies of ceritinib and alectinib was not available in crizotinib-treated patients. In addition, more patients in the ceritinib treatment group had received prior chemotherapy in this study. However, this factor has been clarified by a Cox regression analysis, not fully but to a certain amount, as it was not associated with the treatment efficacy of ceritinib and alectinib. Furthermore, an alternative approach as earlier described, the propensity-score-matched analysis [23], was used to moderate this bias between the alectinib and ceritinib groups and confirmed the finding.

## Conclusion

This analysis demonstrated the reasonable efficacy of second-generation ALK inhibitors in crizotinib-pretreated ALK-positive NSCLC patients. Treatment with alectinib showed higher CNS protection as well as higher PFS in patients in whom crizotinib treatment failed due to intolerance. Both alectinib and ceritinib showed manageable toxicity profiles, with no new signals of adverse effects.

## Data Availability

The datasets generated and/or analysed during the current study are not publicly available because of the local regulation to medical confidentiality but are available from the corresponding author on reasonable request.

## Abbreviations

AEs: Adverse events
ALK: Anaplastic lymphoma kinase
CR: Complete response
CNS: Central nervous system
ECOG: Eastern Cooperative Group performance status
HR: Hazard ratio
NSCLC: Non-small cell lung cancer
OS: Overall survival
PFS: Progression free survival
PR: Partial response
